# Lower Masticatory Performance Is a Risk for the Development of the Metabolic Syndrome: The Suita Study

**DOI:** 10.3389/fcvm.2021.752667

**Published:** 2021-11-26

**Authors:** Shuri Fushida, Takayuki Kosaka, Michikazu Nakai, Momoyo Kida, Takashi Nokubi, Yoshihiro Kokubo, Makoto Watanabe, Yoshihiro Miyamoto, Takahiro Ono, Kazunori Ikebe

**Affiliations:** ^1^Department of Prosthodontics, Gerodontology and Oral Rehabilitation, Osaka University Graduate School of Dentistry, Suita, Japan; ^2^Center for Cerebral and Cardiovascular Disease Information, National Cerebral and Cardiovascular Center, Suita, Japan; ^3^Osaka University, Suita, Japan; ^4^Department of Preventive Cardiology, National Cerebral and Cardiovascular Center, Suita, Japan; ^5^Open Innovation Center, National Cerebral and Cardiovascular Center, Suita, Japan; ^6^Division of Comprehensive Prosthodontics, Faculty of Dentistry & Graduate School of Medical and Dental Sciences, Niigata University, Niigata, Japan

**Keywords:** geriatric dentistry, prosthodontics, mastication, epidemiology, preventive dentistry, cardiovascular diseases

## Abstract

**Objectives:** Declined masticatory function has recently been receiving attention as a risk factor for poor general health. The present longitudinal analysis was conducted to clarify the relationship between decreased masticatory performance and the development of the metabolic syndrome (MetS) in a general urban cohort in Japan.

**Methods:** We surveyed 599 participants (254 men, 345 women; mean age at baseline, 65.8 ± 7.8 years) who underwent physical health checkups in the Suita study. We evaluated masticatory performance at baseline using test gummy jelly and divided participants into two groups: a “Lower group,” comprising participants in the lower 25% of the masticatory performance at baseline; and a “Normal group,” comprising all others. We estimated hazard ratios (HRs) for the Lower group by using Cox proportional hazard regression analysis to develop the MetS and the components of the MetS at follow-up, adjusting for age, smoking status, and periodontal status.

**Results:** On Cox proportional hazard regression analysis, the multivariable adjusted hazard ratio for the development of the MetS in the Lower group was 2.24 (95% confidence interval, 1.12–4.50) in men. The multivariable adjusted hazard ratio for the development of high blood pressure was 3.12 (1.42–6.87), for high triglycerides was 2.82 (1.18–6.76), and for high fasting plasma glucose was 2.65 (1.00–7.00) in men.

**Conclusions:** Lower masticatory performance suggested to be a risk factor for the development of the MetS as well as MetS components such as high blood pressure, high triglycerides, and high fasting plasma glucose in Japanese men.

## Introduction

Metabolic syndrome (MetS) is a complex condition of overlapping hypertension, abdominal obesity, dyslipidemia, and hyperglycemia in the same individual, and it has been reported as a risk factor for the development of cardiovascular diseases (1). Since the World Health Organization (WHO) published the concept of the MetS in 1999 (2), its prevention has been emphasized, and various measures have been taken around the world (3, 4). Nevertheless, the prevalence of the MetS is still increasing worldwide, affecting an estimated one-quarter of the global population (5). Previously, sex (6), educational level (6), smoking habits (7), eating habits (8), and exercise habits (9) were reported as factors related to the MetS. However, the MetS is a complex pathological condition caused by the various effects of these factors, and there may be factors that have not yet been clarified. Therefore, identifying the risk factors affecting the MetS is thus a major goal for preventing cardiovascular diseases.

Many studies have reported that chronic inflammation from periodontal diseases affects the MetS (10). On the other hand, some reports have shown that the changes in dietary habits and nutritional intake resulting from the declined masticatory function accompanying tooth loss affect the MetS (11). It has been reported that factors related to masticatory function, such as tooth loss (11) and decreased occlusal support (12), are associated with the MetS. However, few studies have shown the relationship between objective oral function and the MetS (13).

Evaluating masticatory performance is one method that reflects oral function objectively (14). Previous studies have reported that tooth loss, lower occlusal force (15), and progression of periodontal disease (16) lead to decreased masticatory performance. Furthermore, some studies have reported that lower masticatory performance is related to diabetes mellitus (17) and obesity (18) through changes in nutritional intake. Lower masticatory performance may thus be related to the development of the MetS. Kikui et al. have already reported a relationship between masticatory performance as evaluated using test gummy jelly and the incidence of the MetS in a general urban Japanese population (13). However, they used a cross-sectional study design and could not make any definitive comment on the longitudinal relationship between masticatory performance and the development of the MetS.

The present study therefore aimed to evaluate the hypothesis that lower masticatory performance at baseline represented a risk factor for the future development of the MetS in a general urban Japanese population in a longitudinal study.

## Materials and Methods

### Study Participants

**Figure 1:**
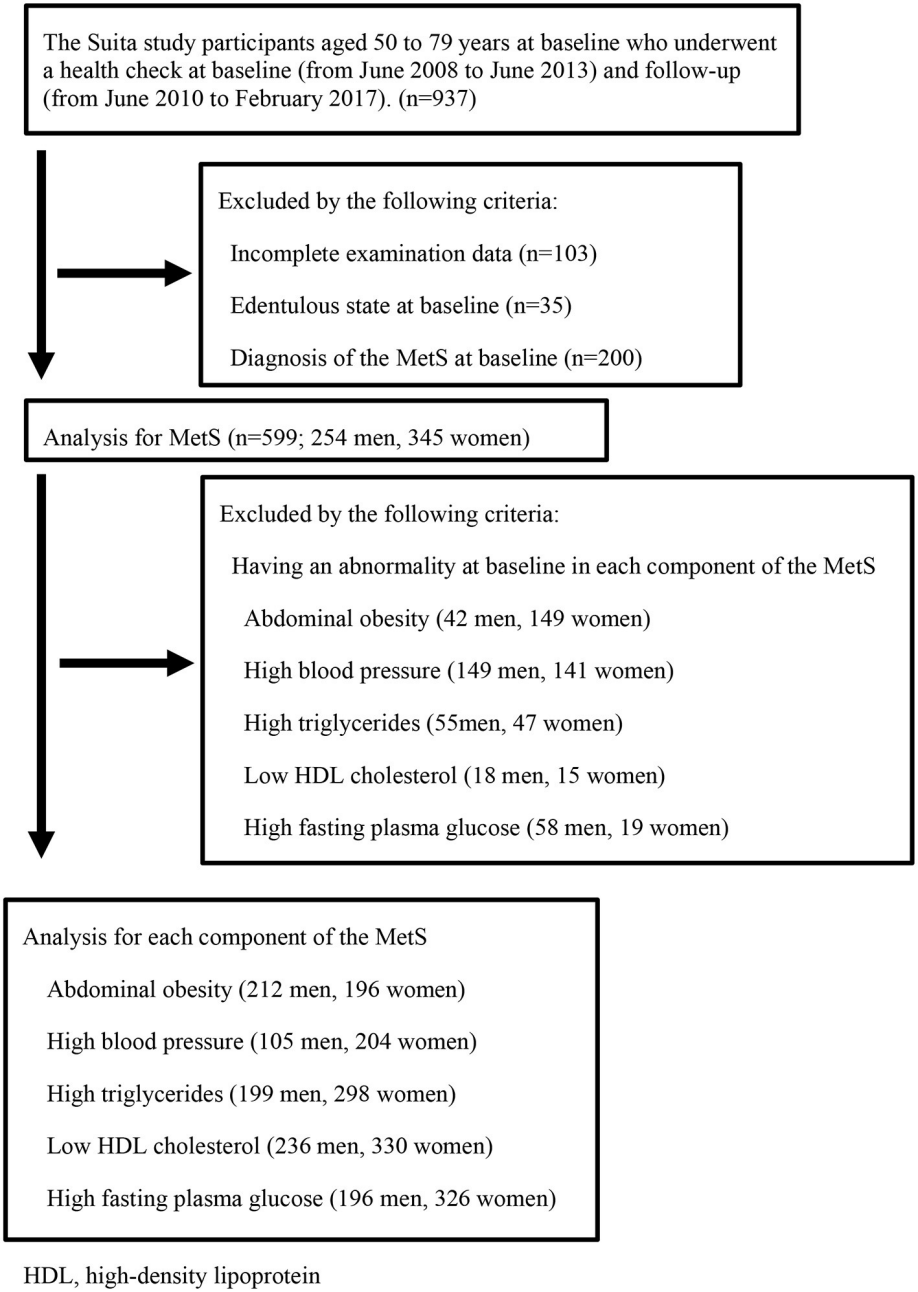
Flow-chart of study participants. HDL, high-density lipoprotein.

Participants in this study were the general urban population of Suita city, Osaka prefecture, Metropolitan prefecture, Japan, who underwent physical health checkups in the Suita study, a cardiovascular disease cohort study conducted by the Department of Preventive Cardiology of the National Cerebral and Cardiovascular Center (19). We recruited 937 individuals who underwent a health check at both baseline (from June 2008 to June 2013) and follow-up (from June 2010 to February 2017). Of these, 338 individuals were excluded due to incomplete data (*n* = 103), an edentulous state at baseline (*n* = 35), or diagnosis of the MetS at baseline (*n* = 200). Finally, 599 participants (254 men, 345 women) were included in the study (Figure 1).

The ethical committee approved the study protocol of the National Cerebral and Cardiovascular Center (approval number M19-062-4, M25-032-2), and only individuals who had given consent after receiving full written and oral explanations of the study were evaluated.

### Medical Examinations

All participants were instructed to fast for 12 h before each health check. Routine blood tests were performed, including triglycerides, high-density lipoprotein (HDL), and fasting blood glucose. Waist circumference, systolic blood pressure (SBP), and diastolic blood pressure (DBP) were measured.

The MetS was defined using National Cholesterol Education Program's Adult Treatment Panel III (NCEP-ATPIII) criteria (20). The five components of the MetS were defined as follows: high blood pressure, SBP ≥130 mmHg and/or DBP ≥85 mmHg and/or taking antihypertensive medicines; high blood glucose level, fasting blood glucose ≥110 mg/dL and/or taking diabetic medicines; hypertriglyceridemia, triglycerides ≥150 mg/dL and/or taking antilipidemic medicines; low HDL-cholesterol, HDL-cholesterol <40 mg/dL in men or <50 mg/dL in women; and abdominal obesity, waist circumference (in the Asian diagnostic criteria) ≥90 cm in men or ≥80 cm in women. The MetS was diagnosed based on the presence of ≥3 of these 5 components.

Participants were surveyed regarding lifestyle using questionnaires. Smoking status was divided into “never smoking,” “quit smoking,” and “current smoking.” Participants who responded “current smoking” were defined as smokers.

### Oral Examinations

#### Periodontal Tissue Examination

Periodontal tissue examination was carried out with participants in a supine position on a bed under artificial lighting of sufficient brightness. Periodontal status was evaluated on the basis of the Community Periodontal Index (CPI) (21). The teeth examined were the maxillary and mandibular left and right first and second molars, the maxillary right central incisor, and the mandibular left central incisor, for a total of 10 teeth. When this examination was not possible because the incisor to be examined was missing, the same tooth on the opposite side was examined. No evaluation was carried out when all the teeth to be surveyed were lost. Periodontal status was examined using a CPI probe (Periodontal Probe; YDM, Tokyo, Japan) at six points in the periodontal pocket of each tooth according to the following CPI code criteria, with the highest code value recorded: Code 0, no finding of gingival inflammation; Code 1, bleeding found after probing; Code 2, dental calculus deposits (including those detected by probing up to 4 mm beneath the gingival margin); Code 3, periodontal pocket depth ≥4 mm but <6 mm; and Code 4, periodontal pocket depth ≥6 mm. In this study, CPI Code ≥3 was defined as “with periodontitis,” and CPI Code 0–2 was defined as “without periodontitis.”

#### Masticatory Performance Examination

Participants were instructed to chew a piece of test gummy jelly (Soshaku-noryoku sokuteiyou gummy jelly; UHA Mikakuto, Osaka, Japan) using 30 chewing strokes on the preferred side, and to expectorate the comminuted jelly onto a piece of cotton gauze spread over the top of a paper cup, without leaving any gummy particles in the mouth. The cotton gauze and comminuted pieces were then rinsed under running water for 30 s to remove as much saliva and glucose adhering to the surfaces as possible. The comminuted jelly alone was subsequently placed in a plastic container, and water (35°C, 15 mL) was injected into the container. The contents were agitated for 10 s at 400 rpm with a magnetic stirrer (PC-410D Digital Stirrer; Corning Incorporated, New York, USA). Immediately after agitation, a small amount of the supernatant was collected using a set of forceps and placed in contact with the tip of a sensor fitted to a commercially available instrument for self-monitoring of blood glucose (Glutest Every; Sanwa Kagaku Kenkyusho, Nagoya, Japan), and the glucose concentration (mg/dL) displayed after 15 s was recorded. The increase in surface area of the comminuted jelly (mm^2^) (y) was calculated from the glucose concentration (x) using the regression formula y = 15x-250, and this was regarded as masticatory performance. For participants who wore removable dentures, masticatory performance was measured with the dentures in place. Participants were surveyed the presence or absence of pain in the temporomandibular joint when masticatory performance examinations were performed. We excluded the participants who answered they had pain.

In this study, participants in the lower 25% of the masticatory performance at baseline by sex were defined as the “Lower group,” and all others were defined as the “Normal group” (22).

### Statistical Analysis

We conducted analyses in this study by sex (1), because of the differences between the sexes in the risk factors for the MetS (6). First, we compared each variable between groups of masticatory performance using the Student's *t*-test for continuous variables and the chi-squared test for categorical variables. We used the Shapiro-Wilk test for masticatory performance (men: *p* = 0.057, women: *p* = 0.274) and Q-Q plot, and confirmed that the data had a normal distribution.

In this study, as the first-stage analysis, the participants were limited to those who did not have MetS at baseline (*n* = 599) to assess the relationship between lower masticatory performance and MetS (Figure 1). We estimated hazard ratios (HRs) to develop the MetS at follow-up in the Lower group using Cox proportional hazard regression, adjusting for age, smoking status, and periodontal status. Additionally, as a second-stage analysis, we assessed lower masticatory performance had a large effect which of the components of the MetS (abdominal obesity, high blood pressure, high triglycerides, low HDL cholesterol, high fasting plasma glucose). This second-stage analysis was performed by excluding those who had each component at baseline from the participants who were analyzed in the first stage. The number of participants analyzed was as follows: abdominal obesity, *n* = 408; high blood pressure, *n* = 309; high triglycerides, *n* = 497; low HDL cholesterol, *n* = 566; and high fasting plasma glucose, *n* = 522. We estimated HRs to develop each component of the MetS at follow-up in the Lower group using Cox proportional hazard regression, adjusting for age, smoking status, and periodontal status. Values of *p* <0.05 were considered significant for all analyses. All statistical analyses were performed using IBM SPSS Statistics version 25 (SPSS Japan, Tokyo, Japan).

## Results

The mean age at baseline was 65.8 ± 7.8 years, and the mean follow-up was 4.4 ± 1.3 years. During follow-up, 88 participants (50 men, 38 women) developed the MetS.

Participants' characteristics at baseline according to the two groups of masticatory performance are shown in Table 1. In men, the Lower group was significantly older and showed higher fasting blood glucose levels than the Normal group. In women, the Lower group had significantly poor periodontal status than the Normal group.

**Table 1:** Characteristics of the study population by masticatory performance and sex.

	**Men**		**Women**	
	**Normal group**	**Lower group**	**Normal group**	**Lower group**
*n*	191	63	259	86
Masticatory performance^a^, mm^2^	5615 ± 1523	2170 ± 880	5267 ± 1310	2185 ± 816
Age^a^, y	65.5 ± 7.7	70.1 ± 6.3^*^	64.7 ± 7.7	66.5 ± 8.3
Waist circumference^a^, cm	84.6 ± 7.0	83.9 ± 6.4	78.9 ± 8.2	79.9 ± 9.0
SBP^a^, mmHg	127.2 ± 17.1	129.0 ± 16.5	122.5 ± 19.5	124.1 ± 18.9
DBP^a^, mmHg	79.7 ± 10.5	79.6 ± 8.9	74.3 ± 11.0	75.6 ± 9.2
Triglycerides^a^, mg/dL	101.7 ± 63.2	100.5 ± 54.5	84.9 ± 33.4	80.8 ± 34.3
HDL-cholesterol^a^, mg/dL	57.5 ± 14.6	55.8 ± 15.0	70.6 ± 13.9	69.1 ± 15.9
Fasting blood glucose^a^, mg/dL	104.0 ± 12.8	109.0 ± 23.5^*^	97.8 ± 7.9	96.6 ± 6.7
Current smoking^b^, %	19.4	25.4	3.9	3.5
Periodontitis^b^, %	53.4	58.7	37.8	54.7^*^

*Values are given as means ± SD or frequencies (%)*.

*The Lower group was defined as ≤ 25% of masticatory performance*.

*SBP, systolic blood pressure; DBP, diastolic blood pressure; HDL, high-density lipoprotein*.

*Periodontitis was defined as CPI ≥ code 3*.

a *Student's t-test*.

b *Chi-squared test*.

* *Difference between Normal and Lower groups, p < 0.05*.

The results of Cox proportional hazard regression analysis showed that the HRs to develop the MetS in the Lower group in men were 2.03 [95% confidence interval (CI), 1.01–4.05] in the age-adjusted model and 2.24 (1.12–4.50) in the multivariatble-adjusted model (Table 2). No significant association between masticatory performance and development of the MetS was seen in either the age-adjusted model or the multivariable-adjusted model in women.

**Table 2:** Multivariable-adjusted hazard ratios (95% CI) for the MetS by masticatory performance.

	**Masticatory performance**	
	**Normal group**	**Lower group**
**Men (*****n*** **=** **254)**		
Age-adjusted	1 (Ref)	2.03 (1.01–4.05)
Multivariable-adjusted	1 (Ref)	2.24 (1.12–4.50)
**Women (*****n*** **=** **345)**		
Age-adjusted	1 (Ref)	1.20 (0.54–2.69)
Multivariable-adjusted	1 (Ref)	1.14 (0.51–2.57)

*CI, confidence interval; Ref, reference*.

*The Lower group was defined as ≤ 25% of masticatory performance*.

*Multivariable hazard ratios were estimated adjusting for age, smoking status, and periodontitis*.

During follow-up, 81 participants (39 men, 42 women) developed high blood pressure, 69 (29 men, 40 women) developed high triglycerides, 25 (11 men, 14 women) developed low HDL cholesterol, 42 (26 men, 16 women) developed high fasting plasma glucose, and 69 (31 men, 38 women) developed abdominal obesity.

The results of Cox proportional hazard regression analysis showed that HRs for the development of high blood pressure in the Lower group in men were 2.62 (1.25–5.52) in the age-adjusted model and 3.12 (1.42–6.87) in the multivariable-adjusted model (Table 3). The results of Cox proportional hazard regression analysis showed that HRs for the development of high triglycerides in the Lower group in men were 2.74 (1.13–6.68) in the age-adjusted model and 2.82 (1.18–6.76) in the multivariable-adjusted model. The results of Cox proportional hazard regression analysis showed that HRs for the development of high fasting plasma glucose in the Lower group in men were 2.49 (0.97–6.37) in the age-adjusted model and 2.65 (1.00–7.00) in the multivariable-adjusted model.

**Table 3:** Multivariable-adjusted hazard ratios (95% CI) for the MetS components by masticatory performance in men.

	**Masticatory performance**	
	**Normal group**	**Lower group**
**Abdominal obesity (*****n*** **=** **212)**		
Age-adjusted	1 (Ref)	1.42 (0.60–3.36)
Multivariable-adjusted	1 (Ref)	1.96 (0.84–4.59)
**High blood pressure (*****n*** **=** **105)**		
Age-adjusted	1 (Ref)	2.62 (1.25–5.52)
Multivariable-adjusted	1 (Ref)	3.12 (1.42–6.87)
**High triglycerides (*****n*** **=** **199)**		
Age-adjusted	1 (Ref)	2.74 (1.13–6.68)
Multivariable-adjusted	1 (Ref)	2.82 (1.18–6.76)
**Low HDL cholesterol (*****n*** **=** **236)**		
Age-adjusted	1 (Ref)	2.06 (0.48–8.79)
Multivariable-adjusted	1 (Ref)	1.83 (0.30–11.36)
**High fasting plasma glucose (*****n*** **=** **196)**		
Age-adjusted	1 (Ref)	2.49 (0.97–6.37)
Multivariable-adjusted	1 (Ref)	2.65 (1.00–7.00)

*CI, confidence interval; Ref, reference*.

*The Lower group was defined as ≤ 25% of masticatory performance*.

*Multivariable hazard ratios were estimated adjusting for age, smoking status, and periodontitis*.

No significant association between masticatory performance and the development of MetS components was seen in either the age-adjusted model or the multivariable-adjusted model in women (Table 4).

**Table 4:** Multivariable-adjusted hazard ratios (95% CI) for MetS components by masticatory performance in women.

	**Masticatory performance**	
	**Normal group**	**Lower group**
**Abdominal obesity (*****n*** **=** **196)**		
Age-adjusted	1 (Ref)	1.84 (0.84–4.04)
Multivariable-adjusted	1 (Ref)	1.39 (0.63–3.08)
**High blood pressure (*****n*** **=** **204)**		
Age-adjusted	1 (Ref)	1.11 (0.49–2.55)
Multivariable-adjusted	1 (Ref)	1.28 (0.55–2.97)
**High triglycerides (*****n*** **=** **298)**		
Age-adjusted	1 (Ref)	1.55 (0.74–3.23)
Multivariable-adjusted	1 (Ref)	1.85 (0.88–3.92)
**Low HDL cholesterol (*****n*** **=** **330)**		
Age-adjusted	1 (Ref)	2.60 (0.85–7.96)
Multivariable-adjusted	1 (Ref)	1.42 (0.43–4.73)
**High fasting plasma glucose (*****n*** **=** **326)**		
Age-adjusted	1 (Ref)	0.73 (0.20–2.59)
Multivariable-adjusted	1 (Ref)	0.78 (0.22–2.79)

*CI, confidence interval; Ref, reference*.

*The Lower group was defined as ≤ 25% of masticatory performance*.

*Multivariable hazard ratios were estimated adjusting for age, smoking status, and periodontitis*.

## Discussion

To the best of our knowledge, this study is the first to show relationships between objective masticatory performance and the development of the MetS through a longitudinal study of a general urban population. The findings showed that lower masticatory performance might be a risk factor for the development of the MetS in men.

Mean masticatory performance in the Lower group was 2,170 ± 830 mm^2^ in men and 2,185 ± 816 mm^2^ in women. Kosaka et al. reported that the mean masticatory performance in a lost occlusal support group (Eichner B4 and C1-3) was 2,439 ± 1,671 mm^2^ (23). Furthermore, the participants in the lost occlusal support group reportedly showed lower intakes of foods that were difficult to chew, such as vegetables and fruit (24). Participants in the present Lower group may thus have had similar masticatory performance to individuals who had lost occlusal support, and so they may have been exposed to similar adverse effects in terms of nutritional intake.

The results of Cox proportional hazard regression analysis showed a significant association between masticatory performance and development of the MetS in men. This association was identified after adjusting for risk factors of the MetS such as smoking (7) and periodontitis (10), all of which have been reported as significantly related to the MetS in many prior studies. Furthermore, according to similar Cox proportional hazard regression analyses, masticatory performance was significantly associated with the development of high blood pressure, high triglycerides, and high fasting plasma glucose in men. As a background to these results, lower masticatory performance may lead to changes in dietary habits and nutritional intake, and it may cause adverse effects on general health. Inomata et al. reported that masticatory function was associated with intakes of vitamins A, C, and B4, folate, and dietary fiber, and they considered that lower masticatory performance might restrict the choice of foods (25). Furthermore, Iwasaki et al. reported that intakes of minerals, vitamins A and C, and dietary fiber were decreased in cases of reduced occlusal support in a 5-year follow-up study (26). Vitamins A and C have effects in preventing diabetes mellitus and cardiovascular disease due to their antioxidant effects, and these intakes are thus associated with the MetS (27). Increased intakes of dietary fiber cause reductions in blood pressure and blood glucose (28). With higher intakes of vegetables, fruit, fish, and natto (fermented soy beans), the prevalence of the MetS decreases (29). On the other hand, tooth loss can reportedly cause increased carbohydrate intake and elevated blood glucose levels (24). Lower masticatory performance may therefore restrict foods that can be chewed and cause decreases in intakes of dietary fiber and vitamins and increased carbohydrate intake. Based on these reports and the results of the present study, imbalances in nutrition may exert complex effects on blood pressure, blood glucose levels, and serum lipid levels, and the subsequent development of the MetS.

No significant associations were found between masticatory performance and the development of low HDL cholesterol and abdominal obesity. HDL cholesterol has the role of transporting increased triglycerides to the liver and promoting triglyceride metabolism. Therefore, a decrease in HDL cholesterol is a phenomenon that occurs after the increase in triglycerides, and the change during the follow-up period of 4.4 ± 1.3 years on average might be small. On the other hand, it has been reported that lack of exercise and excessive energy intake significantly affect obesity (18). Exercise habits or energy intake were not examined in this study, and their effects, which could thus not be considered in this study, might have affected the results.

In the present study, no significant association was found between masticatory performance and the development of the MetS in women. Geer and Shen reported that women after menopause showed increased insulin resistance and decreased lipid metabolism (30). Furthermore, women after menopause have higher blood pressures, triglyceride levels, and blood glucose levels and lower HDL-cholesterol levels than before menopause (31). The changing hormone balance after menopause may thus affect the development of the MetS. In a study of 4,683 Japanese women, Amagai et al. reported that menopause in Japanese women occurs from around 45 to 54 years of age (32). Because most female participants in the present study were post-menopausal, the changing hormone balance may have affected the results. Furthermore, women may have more careful dietary habits (33) and cook more frequently than men in Japan (34). Women may thus maintain intakes of foods that are difficult to chew by cooking with ingenuity and may show more well-balanced dietary habits. Because of these reports, we found no association between lower masticatory performance and the development of the MetS in women.

The results of the present study suggested that lower masticatory performance is an independent risk factor for the development of the MetS. A previous study reported that masticatory performance decreases with aging (35). On the other hand, prosthodontic treatment can improve the masticatory performance (36, 37) and use of dental services can prevent decreased masticatory performance (38). From the results of the present study, preventing the lower in masticatory performance by prosthodontic treatment and use of dental services may contribute to preventing the development of the MetS. Furthermore, preventing the MetS can contribute to preventing the onset of cardiovascular disease, because the MetS is a risk factor for cardiovascular disease (1). The present findings may offer a new approach for preventing the development of the MetS and cardiovascular disease.

This study had several limitations. The first was that the status of nutrient intakes was not be examined in spite of that lower masticatory performance affected the development of the MetS via adverse effects on nutrient intake remains hypothetical. Second, it was not possible to adjust for other risk factors for the development of the MetS such as level of education, economic status (39), exercise habits, and dietary habits (34). These factors could not be examined in this study, and they may have affected the results. We selected confounding factors based on a previous article assessing the risk factor the development of cardiovascular disease in Japanese (40), because the MetS is a risk factor for the development of cardiovascular disease. Age, sex, and smoking habit were common risk factors for the development of cardiovascular disease in Japanese. Furthermore, in this study, since the analysis was performed by sex, confounding factors were limited due to the sample size. Therefore, we selected age and smoking habit as confounding factors in this study.

## Conclusion

In this study, lower masticatory performance was associated with the development of the MetS in men after adjusting for confounding factors. Furthermore, lower masticatory performance was associated with the development of high blood pressure, high triglycerides, and high fasting plasma glucose in men. Improving and maintaining masticatory performance may offer a new approach to preventing the development of the MetS. The findings of this study will provide a basis for new preventive strategies against the development of the MetS and subsequent cardiovascular diseases.

## Data Availability Statement

The datasets presented in this article are not readily available because request for date disclosure will be granted at the discretion of the Facility Ethics Committee. Requests to access the datasets should be directed to Takayuki Kosaka, kosaka@dent.osaka-u.ac.jp.

## Ethics Statement

The studies involving human participants were reviewed and approved by the National Cerebral and Cardiovascular Center. The patients/participants provided their written informed consent to participate in this study.

## Author Contributions

SF contributed to conception, design, data interpretation, performed statistical analyses, drafted, and critically revised the manuscript. TK contributed to conception, design, data acquisition and interpretation, drafted, and critically revised the manuscript. MN contributed to data interpretation, performed statistical analyses, and critically revised the manuscript. MK and YK contributed to data acquisition and interpretation and critically revised the manuscript. TN, TO, and KI contributed to conception, design, and critically revised the manuscript. MW contributed to data acquisition. YM contributed to conception, design, data acquisition, and critically revised the manuscript and supervision. All authors contributed to the article and approved the submitted version.

## Funding

This study was supported by grants-in-aid from the Ministry of Education, Culture, Sports, Science and Technology of Japan (Nos. 20390489, 23390441, 26293411, 17H04388, and 19K19123) and internal research grants from the National Cerebral and Cardiovascular Center (22-4-5 and 27-4-3).

## Conflict of Interest

The authors declare that the research was conducted in the absence of any commercial or financial relationships that could be construed as a potential conflict of interest.

## Publisher's Note

All claims expressed in this article are solely those of the authors and do not necessarily represent those of their affiliated organizations, or those of the publisher, the editors and the reviewers. Any product that may be evaluated in this article, or claim that may be made by its manufacturer, is not guaranteed or endorsed by the publisher.
