# Corrigendum: Lower Masticatory Performance Is a Risk for the Development of the Metabolic Syndrome: The Suita Study

**DOI:** 10.3389/fcvm.2022.872326

**Published:** 2022-03-03

**Authors:** Shuri Fushida, Takayuki Kosaka, Michikazu Nakai, Momoyo Kida, Takashi Nokubi, Yoshihiro Kokubo, Makoto Watanabe, Yoshihiro Miyamoto, Takahiro Ono, Kazunori Ikebe

**Affiliations:** ^1^Department of Prosthodontics, Gerodontology and Oral Rehabilitation, Osaka University Graduate School of Dentistry, Suita, Japan; ^2^Center for Cerebral and Cardiovascular Disease Information, National Cerebral and Cardiovascular Center, Suita, Japan; ^3^Osaka University, Suita, Japan; ^4^Department of Preventive Cardiology, National Cerebral and Cardiovascular Center, Suita, Japan; ^5^Open Innovation Center, National Cerebral and Cardiovascular Center, Suita, Japan; ^6^Division of Comprehensive Prosthodontics, Faculty of Dentistry & Graduate School of Medical and Dental Sciences, Niigata University, Niigata, Japan

**Keywords:** geriatric dentistry, prosthodontics, mastication, epidemiology, preventive dentistry, cardiovascular diseases

In the original article, the **Funding** statement is missing the grant number: 19K19123. The corrected statement appears below.

**Funding:**

“This study was supported by grants-in-aid from the Ministry of Education, Culture, Sports, Science and Technology of Japan (Nos. 20390489, 23390441, 26293411, 17H04388, and 19K19123) and internal research grants from the National Cerebral and Cardiovascular Center (22-4-5 and 27-4-3).”

The authors apologize for this error and state that this does not change the scientific conclusions of the article in any way. The original article has been updated.

## Publisher's Note

All claims expressed in this article are solely those of the authors and do not necessarily represent those of their affiliated organizations, or those of the publisher, the editors and the reviewers. Any product that may be evaluated in this article, or claim that may be made by its manufacturer, is not guaranteed or endorsed by the publisher.

